# Evolution of NO_2_ levels in Spain from 1996 to 2012

**DOI:** 10.1038/srep05887

**Published:** 2014-07-30

**Authors:** Carlos A. Cuevas, Alberto Notario, José Antonio Adame, Andreas Hilboll, Andreas Richter, John P. Burrows, Alfonso Saiz-Lopez

**Affiliations:** 1 Atmospheric Chemistry and Climate Group. Institute of Physical Chemistry Rocasolano, CSIC, Madrid, Spain; 2 Departamento de Química Física. Facultad de Ciencias Químicas, Universidad de Castilla-La Mancha, Ciudad Real, Spain; 3 Atmospheric Sounding Station-El Arenosillo. National Institute for Aerospace Technology (INTA), Atmospheric Research and Instrumentation Branch. Mazagón-Huelva, Spain; 4 Institute of Environmental Physics and Remote Sensing, University of Bremen, Bremen, Germany

## Abstract

We report on the evolution of tropospheric nitrogen dioxide (NO_2_) over Spain, focusing on the densely populated cities of Barcelona, Bilbao, Madrid, Sevilla and Valencia, during 17 years, from 1996 to 2012. This data series combines observations from in-situ air quality monitoring networks and the satellite-based instruments GOME and SCIAMACHY. The results in these five cities show a smooth decrease in the NO_2_ concentrations of ~2% per year in the period 1996–2008, due to the implementation of emissions control environmental legislation, and a more abrupt descend of ~7% per year from 2008 to 2012 as a consequence of the economic recession. In the whole Spanish territory the NO_2_ levels have decreased by ~22% from 1996 to 2012. Statistical analysis of several economic indicators is used to investigate the different factors driving the NO_2_ concentration trends over Spain during the last two decades.

Ozone, fine particulate matter (PM), nitrogen oxides (NO_x_ = NO + NO_2_), and sulfur dioxide (SO_2_) are the most important air pollutants in the troposphere, which represent a serious threat for ecosystems and human health^1^. Although the sources of these pollutants in the European Union (EU) have considerably been reduced in the last ten years, the air quality is still far from the levels required by the EU legislation. The EU environmental policies requirements are currently being reviewed to be adapted to safer levels for human health^2^.

NO_x_ are important in tropospheric chemistry for several reasons, including i) their role in the photochemical production of ozone^3^, ii) their atmospheric processing and subsequent deposition which leads to acidification and eutrophication in ecosystems through the formation of nitric acid (HNO_3_)^4^,^5^, iii) their role as precursors of the nitrate radical (NO_3_) (which controls the oxidizing capacity of the nocturnal atmosphere^6^,^7^), and iv) their effect on the radiative forcing of the atmosphere, directly through the absorption of radiation by NO_2_, or indirectly through ozone formation and altering the lifetimes of several greenhouse gases^8^,^9^. Although NO_x_ are also naturally produced by soil emissions, biomass burning and lighting, their main source is anthropogenic through the combustion of fossil fuels such as coal, oil (mainly in urban traffic) and gas. Hence, NO_x_ emissions in a country or region are directly related to its industrial and economic development, and are consequently affected by changes in the economic and industrial activity, in addition to the implementation of environmental policies^10^,^11^,^12^.

Most of the NO_x_ emissions from combustion systems are in the form of NO, although in diesel vehicles NO_2_ emissions can reach up to 70% of the emitted NO_x_^13^. From 1996 to 2011, the gasoline/gas-oil percentage ratio in Spain has changed from 75/25 to 45/54(1% corresponds to electric vehicles)^14^, therefore a relatively higher amount of NO_x_ emissions is currently produced in the form of NO_2_. As sunlight produces an equilibrium between NO and NO_2_ through a photochemical cycle involving ozone^9^, space-based observations of NO_2_ can be used to locate sources of NO_x_ emissions due to the short lifetime of NO_2_^15^. The first studies about the temporal evolution of tropospheric NO_2_ were reported by Richter et al.^16^ for the 1996–2004 time period in the main industrialized and populated areas of the world, and Irie at al.^17^ for 1996–2002 focusing on East Asia. The first study reporting the effect of the economic downturn in the NO_2_ levels was published by Lin and McElroy^12^, showing a clear decrease in China from late 2008 to late 2009. Since these studies there have been other reports about the evolution of tropospheric NO_2_ as seen from space^18^,^19^,^20^. More recently, the global study on tropospheric NO_2_ trends by Richter et al.^16^ has been updated for the 1996–2011 period by Hilboll et al.^15^. Previous reports of NO_2_ trends have also highlighted a clear reduction in the NO_2_ levels over urban areas due to both the application of anthropogenic NO_x_ emission control policies and the effects of the global economic crisis^10^,^11^,^21^.

In this work we report on the NO_2_ geographical distribution and concentration trends over Spain using combined multiple satellite datasets and ground-based observations from five metropolitan areas in the country: Madrid (3.233.527 inhabitants), Barcelona (1.620.943 inhabitants), Valencia (797.028 inhabitants), Sevilla (702.355 inhabitants) and Bilbao (351.629 inhabitants)^22^. Ground-based observations in León and Ponferrada are also reported, as these relatively small cities are located in the region where satellite measurements detect the higher decrease. We assess the contribution of environmental policies and the recent severe economic recession to the observed reduction in NO_2_ levels over Spain in the last two decades. Finally, the variation of the main economic indicators of the country is compared with the measured NO_2_ levels, to infer the possible relationship between the NO_x_ emissions and the economic evolution of Spain.

## Results

### Satellite-based NO_2_ observations and trends

**Figure 1:**
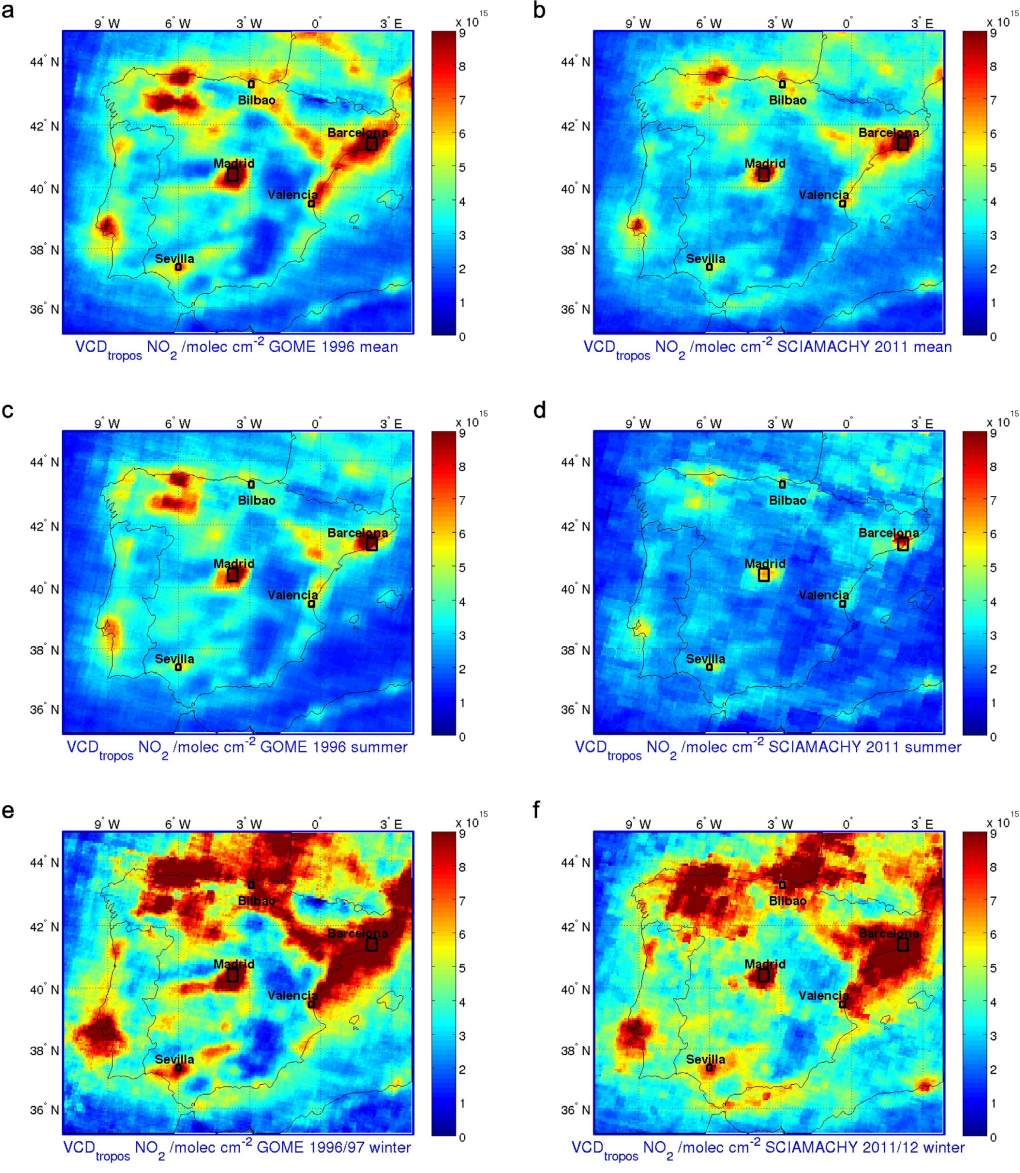
Averaged tropospheric NO_2_ VCD for different seasons during 1996, using the GOME resolution-corrected dataset (a, c and e), and 2011/2012 using SCIAMACHY data (b, d, f). Top: annual averages. Middle: summer (Jun-Jul-Aug) averages. Bottom: winter (Dec-Jan-Feb) averages. Figure created by the authors using Matlab R213a.

Figure 1 shows averaged tropospheric NO_2_ Vertical Column Density (VCD) maps for 1996 and 2011 over the Iberian Peninsula, with data from the GOME and SCIAMACHY instruments, respectively. Due to the different ground pixel size between these different sensors, a resolution correction method is applied to the low spatial resolution data from GOME^15^ (see Methods). This procedure allows building a time-consistent NO_2_ dataset from 1996 to 2012. The figure includes annual, winter (Dec-Jan-Feb), and summer (Jun-Jul-Aug) NO_2_ column averages for the first and last years of the time series. Data from the GOME resolution corrected data product have been used for 1996. The plot for the last year of the series is made from 2011 SCIAMACHY measurements, as the operative cycle of this instrument ended in April 2012. Figure 1 clearly illustrates the difference between concentrations and geographical distribution of tropospheric NO_2_ in 1996 and 2011. The seasonality of NO_2_ values is also evident for both years, showing higher concentrations in winter that contrast with summer values. As expected in mid-latitude developed countries, the lower solar radiation levels and the higher energy consumption, due to building heating systems, produce higher values of tropospheric NO_2_ in the winter season^23^.

The highest levels of NO_2_ are observed in the regions of Madrid and Cataluña, the two largest urban areas of Spain. Madrid's economy is based on the service, construction and industry sectors. Its location in the centre of the country also makes Madrid the main transport knot within the Iberian Peninsula. Cataluña is located in the north-east coast of Spain, and its economy is mainly based on service, tourism and industry. Most of the economic activity in these two regions is located close to the metropolitan areas of the cities of Barcelona and Madrid.

The north and east coastal regions of the peninsula are also areas with considerable industrial activity, and is therefore reflected in high NO_2_ levels (Fig. 1). The north-west part of the peninsula (Asturias, León and Palencia provinces, see Figure S1 for their location) is an important region of mining and power generation industries. Also, considerable maritime transport, mining and steel industries activities take place in the north coast area of the Gulf of Vizcaya, close to the city of Bilbao. At the east coast, most of the economic activity is located in the Valencia region and the Ebro basin, whereas the Guadalquivir basin concentrates most of the economic and industrial activity in the south-west part of Spain, where cities such as Cordoba, Sevilla and Huelva are located.

The reduction in the NO_2_ levels over recent times is evident when comparing the first and last years of the satellite dataset (Fig. 1). The decrease is clear in the annual and summer averages, although the change in winter is less pronounced. Both annual and summer averages show a marked reduction in Madrid, Cataluña, and the north-west part of the peninsula. In winter the drop is less apparent, but still appreciable in Madrid, east coast and north-west areas. Note that the 2011 winter was particularly cold^24^ and thus could be responsible for a higher use of heating systems, incrementing NO_x_ emissions during that winter. The more widespread NO_2_ distributions over the regions of Madrid and Cataluña in 1996 are reduced and limited by 2011 to the metropolitan and localised industrial areas around the cities of Madrid and Barcelona. The decrease in NO_2_ levels and its geographical spread is also clear along the north and east coasts, and Ebro and Guadalquivir basins (Fig. 1).

**Figure 2:**
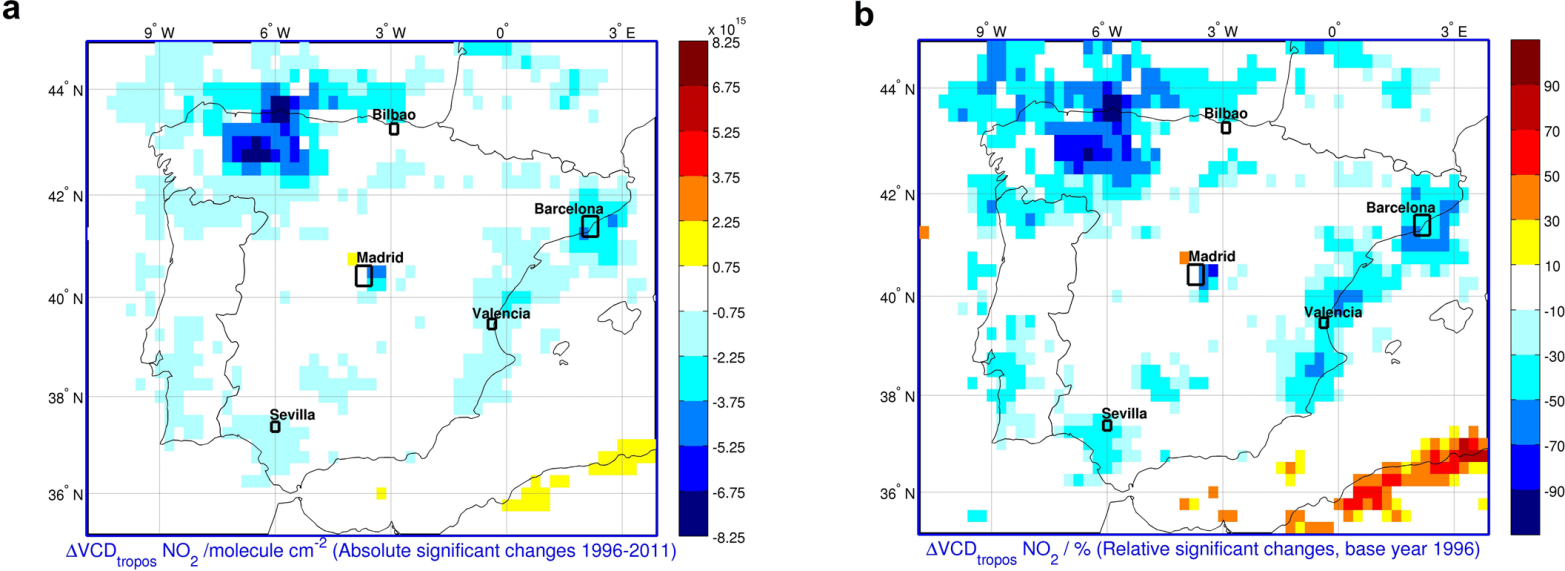
Change in the NO_2_ tropospheric VCD over the Spanish territory derived from the shift trend model for the years 1996–2011 (data with significant trends at the 95% level). Figure created by the authors using Matlab R213a.

To study the time evolution of NO_2_ levels over the Iberian peninsula, the trend model described in Hilboll et al.^15^ has been applied here to the NO_2_ measurements from the GOME and SCIAMACHY instruments. Figure 2 shows the integrated evolution of tropospheric NO_2_ levels during the 1996–2011 time period, including absolute and relative changes. Both plots illustrate the decrease in NO_2_ concentrations over a large part of the peninsula, and particularly in the main economic and industrial areas. NO_2_ levels in the area of Madrid have decreased on an annual average by up to 1.9 × 10^14^ molecules cm^−2^, which adds up to 53% of the levels measured in 1996. This reduction is much higher than the 20% previously reported for a different period (2004–2010) using OMI measurements by Castellanos and Boersma, 2012^10^. This difference is due to the different time period considered and also note the difference in local time at which different instruments overpass Spain. The measurement times for GOME and SCIAMACHY are ~10:30 and ~10:00 local time, while OMI is ~13:45. GOME and SCIAMACHY measurements are closer to the diurnal NO_2_ peak in the early morning, when NO_x_ emissions are more affected by transport sources at the rush hour. Emissions from industry and power plants have a less pronounced diurnal variation; consequently lower NO_2_ VCDs are expected from the OMI measurements resulting in lower relative decreases. These differences in the SCIAMACHY product compared to OMI have been previously studied^25^,^26^,^27^, reporting higher NO_2_ levels from SCIAMACHY in the northern midlatitudes and regions where the NO_x_ emissions come mainly from fossil fuel sources. Other reasons are the different pixel size of OMI and the differences in the instruments and retrieval algorithm. Considerable reductions of ~40, ~36, and ~51% are measured in the areas of Barcelona, Bilbao and Valencia, respectively. In Sevilla, however, a smaller decrease of 27% is observed.

The most widespread and remarkable reduction in NO_2_ levels is detected in the northwest industrial area and the north coast, with the highest decreases in the Asturias region and the provinces of León and Palencia (Fig. 2). These regions have experienced a large reduction in the industrial, mining and coal power plants activities, which together with the application of new emission control technologies have resulted in a drastic reduction in NO_2_ concentrations. The decline in mining and industrial activities in these regions from 2004 to 2009, have led to a fall in the NO_x_ emissions of ~52% from the main power plant stations of the region^28^. A summary of the satellite NO_2_ trends by geographical areas is given in Table 1, including absolute and relative changes.

Recently, Castellanos and Boersma^10^ for Europe and Vrekoussis et al.^21^ for Greece, have also reported a general negative trend in NO_2_ concentrations accelerated since the beginning of the economic recession (i.e., 2008). The trend model can also be applied to the pre-recession and recession time periods, although the nonlinearities in the general trend and the short length of this individual periods yield few significant data. Nevertheless the purpose of this work is to show the evolution of the NO_2_ levels rather than specifically analyse the short time period trends. Overall, from 1996 to 2011, the integrated NO_2_ VCD as observed from space over the entire Spanish territory has been reduced by ~22%.

### NO_2_ trends as measured by *in-situ* air quality networks

**Figure 3:**
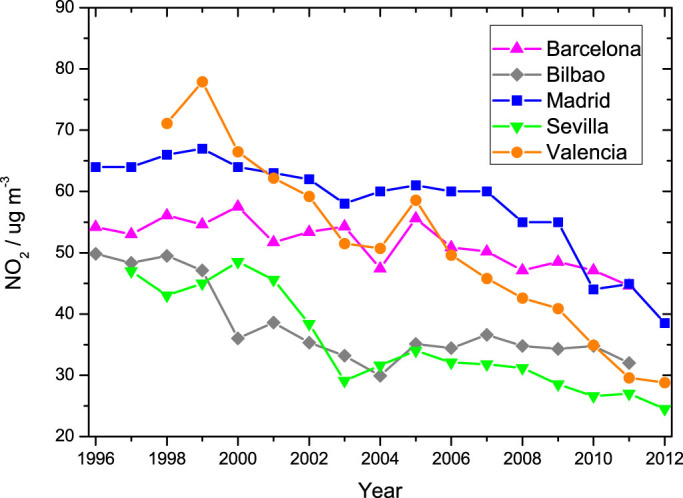
NO_2_ annual averages from air quality monitoring stations.

To quantify the variation of surface NO_2_ levels in the cities of Barcelona, Bilbao, Madrid, Sevilla and Valencia, data from their ground air quality monitoring networks (AQMN) are used. All the *in-situ* monitoring stations employ the chemiluminiscence method described in the Methods section. Figure 3 shows the annually-averaged NO_2_ levels from 1996 to 2012 measured by the available AQMN. All cities show a general decrease in the NO_2_ levels during the last two decades. Regression analysis yields considerable absolute reductions of 67% for Valencia and 52% in Sevilla, while Barcelona exhibits a shallower 18% decrease. Madrid and Bilbao show intermediate decreases of 37% and 35%, respectively. Overall, the average of all cities considered here shows a decrease of 44% in the NO_2_ concentrations.

The results of the statistical regression analysis performed for different cities and time periods are shown in Table 2, including absolute and relative changes. This summary also includes the combined average of all studied cities. Barcelona, Madrid, and Valencia show two well differentiated slopes for the pre-recession and recession periods (−0.5, −0.7, and −3.1/μg m^−3^ yr^−1^ in Barcelona, Madrid and Valencia, respectively, during the pre-recession period, and −0.9, −4.3, and −3.9/μg m^−3^ yr^−1^, respectively, during the recession period). In these three cases the slopes are higher during the recession period. These slopes correspond to annual decreases of 0.9%, 1.1% and 4.3 for Barcelona, Madrid and Valencia, respectively, during the pre-recession period, and 1.9%, 7.8% and 9.1%, respectively, during the economic recession. Sevilla shows similar slopes for both periods, being slightly higher during the pre-recession period (−1.7/μg m^−3^ yr^−1^) than during the economic recession (−1.5/μg m^−3^ yr^−1^). By contrast with the other cities, Bilbao shows a higher slope during the pre-recession period than during the economic recession (−1.4 and −0.8/μg m^−3^ yr^−1^ respectively). Overall, all cities except Bilbao, show higher reductions during the recession period than during the pre-recession. This result suggests an initial reduction driven by the progressive implementation of environmental policies and technology, whereas the second slope can be attributed to the rapid decrease of the economic and industrial activity during the economic downfall between 2008 and 2012.

In order to confirm the pronounced decline in NO_2_ levels reported by the satellite measurements in the North-West region, AQMN data in the cities of Ponferrada and León (province of León) were also investigated. These data show a decrease of 80% in Ponferrada and 66% in León since 1997. Both cities also show a higher decrease during the economic recession period, 72% and 75%, compared to 63% and 41% during the pre-recession period for Ponferrada and León, respectively.

**Figure 4:**
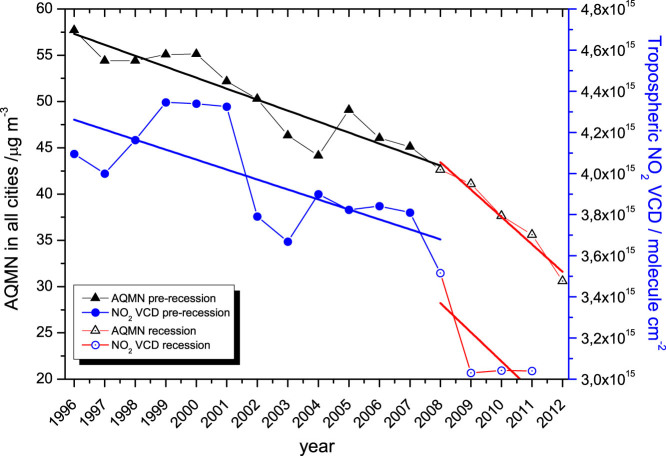
NO_2_ trends in satellite- and ground-based observations during the pre-recession and economic recession periods.

The general annually-averaged NO_2_ trend from both satellite and ground stations is shown in Figure 4. The satellite averages have been made integrating over the entire Spanish territory in the Iberian Peninsula, while the ground stations data correspond to yearly average in the studied cities. Hence the smoother shape in the AQMN dataset, which is more affected by local effects than the satellite measurements. Note that both datasets are not directly comparable, although their combination provides a clear picture about the evolution of NO_2 _levels in Spain during the last two decades.

Both datasets show two distinct slopes corresponding to the pre-recession and recession periods, the slope for the last period being higher. The purpose of this work is to show how the NO_2_ levels have changed in absolute terms during the last two decades, rather than to perform a statistical trend study. Therefore we refer here to ‘general trends', despite the evident variable nonlinear trends observed in the time series. The nonlinearity in the NO_x_ emission trends have already been reported by Konovalov et al.^29^ for the case of Madrid.

## Discussion

**Figure 5:**
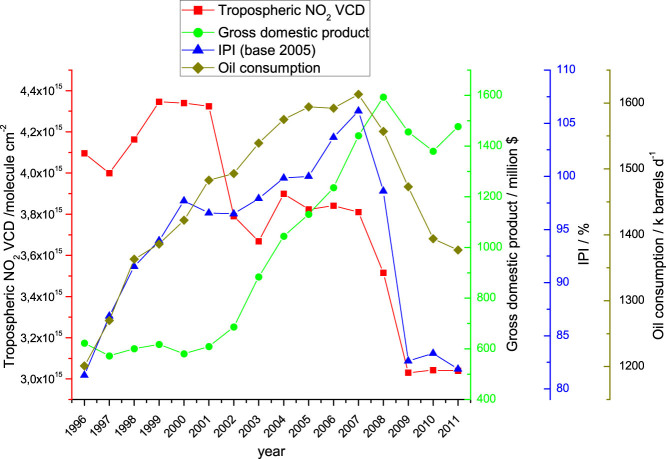
Satellite tropospheric NO_2_ VCD averaged over the Spanish territory, along with the main economic metrics of Spain, Gross Domestic Product (GDP), Industrial Production Index (IPI) and Oil Consumption.

In order to gain insights about how the economic activity affects NO_x_ emissions in Spain, the levels of NO_2_ are compared to the main economic indicators, including Gross Domestic Product (GDP), Industrial Production Index (IPI, base 2005) and Oil Consumption. The GDP is the market value of all officially recognized final goods and services produced within a country in a given period of time. The IPI measures the real production output of manufacturing, mining and utilities of a country. The Oil Consumption refers to the number of barrels consumed in a country per day. NO_x_ natural emissions account for 2.8–3.8% of total emissions in Spain (National Emission Inventory^30^) during the studied period. Therefore the trends in economy and industrial activities can be considered the main source of variation for the NO_x_ emissions in Spain. Bottom-up emissions data from the National Emission Inventory^30^ and the Centre on Emissions Inventories and Projections^31^ are shown in Figures S2 and S3. Note however that the trends in these emission inventories, which follow the trends in the economic parameters of the country, show a discrepancy with both on-ground and satellite data (see Figs. 5, S2 and S3). This highlights potential uncertainties in the trends of emission inventories and suggests that further research is needed to reconcile differences in the trends of observed NO_2_ and the emission inventories during the pre-recession period in Spain.

Although Spain has failed to achieve the Gothenburg protocol targets, it has reduced the NO_x_ emissions by about 20% from 1990 to 2011, as reported by the European Environment Agency^32^. This fact is reflected in the NO_2_ measurements through a reduction by 22% in the NO_2_ levels integrated over Spain as observed from space, and by an average of 45% in the AQMN observations in the five cities studied here. This reduction is considerably higher in the main industrial and economic cores of the country, such as Madrid and the north-west part of the Iberian Peninsula, with reductions of up to 50% in the areas of Madrid and Valencia. In general, there is a correspondence between the tendencies of on-ground and satellite data (Figure 4), having two well differentiated slopes in the trends (i.e. pre-recession and economic recession periods).

On the other hand, the main economic indicators were gradually increasing since 1996 until the beginning of the economic crisis in 2008, when all indicators suddenly fell. These economic metrics refer to the whole country, so satellite NO_2_ VCDs integrated over the whole Spanish territory in the Iberian Peninsula are used to assess the correlation between economic fall and NO_2_ concentrations. In general we find a good correspondence between the economic indicators and the evolution of NO_2_, as shown in Figure 5, despite the 2001–2007 period in which the trends are different. The NO_2_ increase from 1996 to 2000 coincides with a period of economic growth marked by a huge increase of the IPI and a small, but appreciable, increase in the GDP. During this first period it can be considered that the economic rise is directly reflected in the NO_2_ levels. From 2000 onwards the IPI slows down its rising while the NO_2_ VCDs fall from 4.3 × 10^15^ to 3.6 × 10^15^ molecules cm^−2^, and the GDP undergoes a huge and constant growth until the start of the recession in 2008. Consequently, during the period in which the economic activity of the country is rising, the application of environmental policies and the optimization in the combustion processes are likely responsible for the reduction in the emissions of NO_x_ measured from 2000 onwards. Finally during the recession (2008–2012) both indicators and the NO_2_ levels decrease considerably, showing a clear effect of the economy on the levels of anthropogenic pollutants.

The same information can be extracted from the oil consumption evolution in Spain (Figure 5). From 1996 to 2008 it has been growing at constant rate, starting a deep decrease in 2008 with the recession. Note that the 33% increase in the oil consumption from 1996 to 2008 should have led to an increase in the NO_2_ levels during this time period. This indicates the positive effect of emissions legislation upon combustion technologies.

Our results show a lack of correlation between NO_2_ levels and economic indicators from 2001 to 2007, however Vrekousis et al.^21^ reported good agreement between NO_2_ and economic markers in Greece from 2000 to 2011. This difference may be due to the fact that Greece has not reduced its NO_x_ emissions, as a result of environmental legislation, at the same rate as Spain^33^, and hence the better correspondence between economic evolution and NO_x_ emissions in Greece. This reduction in the NO_2_ levels in Spain during the 2001–2007 period, when all the economic factors increase, is a clear signal that the application of environmental policies are efficiently reducing the NO_x_ emissions. This has also been reported for other cities of the European Union as shown by Castellanos and Boersma^10^.

To summarize, the datasets presented here can be divided into two differentiated parts: i) from 1996 to 2008 the application of environmental policies reduced the NO_x_ emissions, with a slight increase from 1996 until 2000 before the effects of environmental policies start to pay off; ii) during the 2008–2012 recession period the economic crisis remarkably reduced the emission even more than the environmental policies. These trends are observable through both datasets, although the absolute changes are higher in the ground-based measurements, showing decreases up to 67% in the city of Valencia. These differences between the two datasets are, as explained in the Methods section, due to the different type of measurements considered. The ground stations represent surface observations within the cities, while the satellite measurements result from integrating the tropospheric column over the whole ground pixel of the instrument, which is larger than the largest of the studied cities. Thus we regard the ground measurements as representative of the local NO_2_ levels and trends in the cities, while the satellite-based observations represent column-integrated NO_2_ levels over larger areas around the cities. The 20% emissions reduction reported by the European Union agree better with the satellite dataset, as these emissions reductions refers to the whole country rather than to specific cities or regions.

The lack of correlation between the economic parameters of Spain and the levels of NO_2_ during the 2001–2007 period, with NO_2_ levels decreasing as the GDP, IP and Oil Consumption grow until 2008, shows that the improvement in the combustion technologies and environmental policies implemented to reduce the nitrogen oxides emissions are paying off. Therefore the air quality is improving, although there is still margin for further improvement as demonstrated by the fact that the daily thresholds of NO_2_ and O_3_ levels are often exceeded in Spanish cities. The steeper slope in the NO_2_ reduction during the recession, when the economy falls, is clear in both datasets. Hence for the case of Spain, still on recession during 2012, it can be expected that the slope of the decrease will recover to the pre-recession values once the economy starts growing again. Finally, this decline in the NO_2_ levels during the last two decades has led to an overall improvement of the air quality in the Iberian Peninsula, and is likely affecting the chemistry of the troposphere in three possible ways: 1) with the reduction in the NO_x_ levels, a variation in the tropospheric ozone levels is also expected, 2) this denoxification of the atmosphere has probably led to lower nitrogen inputs to ecosystems, and 3) the levels and seasonality of HNO_3_ formation as influenced by traffic emissions^34^ have likely been affected.

Finally, it is worth mentioning that future monitoring of air pollution in megacities and industrial regions will greatly benefit from geostationary satellite missions, which will considerably enhance the temporal and spatial resolution of the observations. Therefore it will provide a more accurate tool to verify the effect of implementing environmental policies and to follow the atmospheric impacts of changing emissions.

## Methods

### Description of datasets

Two main datasets including space-based observations and air quality monitoring networks (AQMN) measurements are used to study the evolution of tropospheric NO_2_ over Spain. The satellite observations provide an overview of the NO_2_ geographical distribution and variation not only over major urban areas, but also over the entire Iberian Peninsula, while *in-situ* ground monitoring stations provide insightful information of the pollutant levels evolution within cities. Note that the spatial resolution of current satellite-based spectrometers is still too low to properly assess detailed NO_x_ emissions at a sub-urban scale or from medium-size cities. On the other hand AQMN provide information of pollutants at surface level, while satellite based measurements integrate the NO_2_ vertical column throughout the troposphere (once the stratospheric contribution has been removed). Therefore, both types of measurements are not directly comparable but complementary.

The satellite dataset is based on measurements from the GOME and SCIAMACHY instruments, using the trend model developed by Hilboll et al.^15^. This model accounts for the ground pixel size and instrument differences between GOME and SCIAMACHY, as described below in this section. The *in-situ* dataset has been built using data from the AQMN in each of the cities included in this exercise. Data from a total number of ~55 monitoring stations of urban and sub-urban type have been used during the period of study, with different typology and locations representative of the major emission points within each individual city. These AQMN are operated and maintained by the regional governments of the country, according to current European legislation.

### Surface measurements

The method used to detect NO_2_ in the monitoring station is described in EN 14211:2005 ‘Ambient air quality – Standard method for the measurement of the concentration of nitrogen dioxide and nitrogen monoxide by chemiluminescence, defined by the European Directive (2008/50/CE) as reference method. In this method a pre-reactor separates the NO-NO_2_ reaction from the background chemiluminescence, allowing accurate auto-zeroing of the analyzer. The equipment works under a rigid maintenance program being periodically tested and calibrated.

### Satellite measurements

In this work measurements performed by the satellite based instruments GOME^35^ on board ERS-2, and SCIAMACHY^36^,^37^ on board ENVISAT are employed. The ground pixel of these instruments is 40 × 320 km^2^ and 30 × 60 km^2^ for GOME and SCIAMACHY, respectively. The raw data have been analysed as described in Hilboll et al., 2013a^15^. In short, differential optical absorption spectroscopy^38^ in the 425–450 nm wavelength window^39^ is applied to the measured radiances. The influence of stratospheric NO_2_ has been corrected for using the algorithm detailed in Hilboll et al. 2013^40^, using stratospheric NO_2_ fields from the B3dCTM model^41^,^42^,^43^. Tropospheric air mass factors have been calculated with the radiative transfer model SCIATRAN^44^.

### Correction for the difference in ground pixel size between GOME and SCIAMACHY

To show the spatial distribution of tropospheric NO_2_ columns (see Fig. 1) from GOME measurements, the retrieved data have been post-processed to account for the very low spatial resolution of the GOME instrument. The method is based on spatial averaging of earthshine spectra measured by SCIAMACHY and the extraction of a spatial pattern of the effect of ground pixel size. This effect of ground pixel size is subsequently super-imposed on the original GOME measurements, yielding a data set “GOME res.corr”. In this data set, the magnitude of the tropospheric NO_2_ columns is retrieved from GOME measurements, while the spatial structure is derived from the full SCIAMACHY data set 2003–2011. The method is described in Section 4 of Hilboll et al., 2013^15^.

### Trend model accounting for the difference between GOME and SCIAMACHY measurements

The rate of linear change of tropospheric NO_2_ columns has been calculated using the algorithm described in Section 5 of Hilboll et al., 2013^15^. In short, the trend model explicitly accounts for (a) an offset between the two instruments and (b) for a change in the amplitude of the seasonal variation. The time series of monthly averages of NO_2_ measurements Y (t) is described by the equation 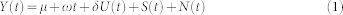
where μ is the VCD_trop_ measurement at time t = 0, *ω* is the monthly trend component, and *t* is the time in months since January 1996. *δ* is the level shift between GOME and SCIAMACHY measurements occurring at time *t* = *T_0_* (which we set to January 2003), and *U(t)* is the step function 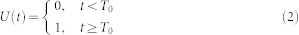
The seasonal component *S(t)* has the same shape (represented by harmonic functions) and varying amplitude for the two instruments, and is modelled by four sine and cosine components, each. The noise process *N(t)* is assumed to be autoregressive with lag 1, and only trends which are significant at a 95% level are considered.

## Author Contributions

C.A.C. and A.S-L. designed research; C.A.C., A.S-L., A.H. and A.R. performed research; C.A.C., A.N., J.A.A., A.H., A.R. and J.P.B. analyzed data; C.A.C., A.S.-L. and A.H. wrote the manuscripts and supplementary information, and all authors reviewed the paper.

## Supplementary Material

Supplementary Information Supplementary information

## Figures and Tables

**Table 1:** Absolute and relative changes in the NO_2_ tropospheric VCD from 1996–2011, derived from the trend model for different geographic regions. (See Table S1 for latitude-longitude coordinates of the studied regions)

Geographic region	Absolute significant changes/molecule cm^−2^	Error/±molecule cm^−2^	Relative significant changes/%	Error/±%
**East coast of Valencia**	−1,27E + 15	3,60E + 14	−33	9
**Ebro Basin and Cataluña**	−1,34E + 15	4,97E + 14	−23	9
**Area of Madrid**	−3,10E + 15	9,48E + 14	−53	16
**Area of Sevilla**	−1,16E + 15	5,33E + 14	−27	12
**Area of Barcelona**	−2,98E + 15	9,89E + 14	−40	13
**Area of Bilbao**	−2,25E + 15	7,29E + 14	−36	12
**North-West**	−2,87E + 15	9,22E + 14	−46	15
**Gulf of Vizcaya**	−1,34E + 15	6,24E + 14	−24	11
**Guadalquivir basin**	−6,36E + 14	3,86E + 14	−17	10
**Area of Valencia**	−2,21E + 15	5,89E + 14	−51	13
**Integrated over Spain**	−9,47E + 14	3,55E + 14	−22	8

**Table 2:** Regression analysis of NO_2_ trends as measured by air quality monitoring stations. Slopes in Table S2

	1996–2012		1996–2008		2008–2012	
City	Decrease/% yr^−1^	Absolute decrease/%	Decrease/% yr^−1^	Absolute decrease/%	Decrease % yr^−1^	Absolute decrease/%
Barcelona	−1.16*	−18.60	−0.92	−11.99	−1.89*	−7.56
Bilbao	−2.19*	−35.02	−2.89	−37.59	−2.27*	−9.08
Madrid	−2.20	−37.45	−1.11	−14.42	−7.84	−39.18
Sevilla	−3.28**	−52.43	−3.64**	−43.66	−4.77	−23.87
Valencia	−4.47	−67.09	−4.32	−47.50	−9.13	−45.66
Average***	−2.64	−44.93	−2.23	−29.02	−6.86	−34.29

*No data available for 2012.

**No data available for 1996.

***Average of the five cities
