# Supplementary material for: Evolution of NO2 levels in Spain from 1996 to 2012
Source: Sci Rep. 2014 Jul 30;4:5887. doi: 10.1038/srep05887 (PMC4115208; doi:10.1038/srep05887)
Supplement: Supplementary Information [file srep05887-s1.pdf]

Evolution of NO_2 levels in Spain from 1996 to 2012

Carlos A. Cuevas, Alberto Notario, José Antonio Adame, Andreas Hilboll,

Andreas Richter, John P. Burrows and Alfonso Saiz-Lopez

Supplementary information

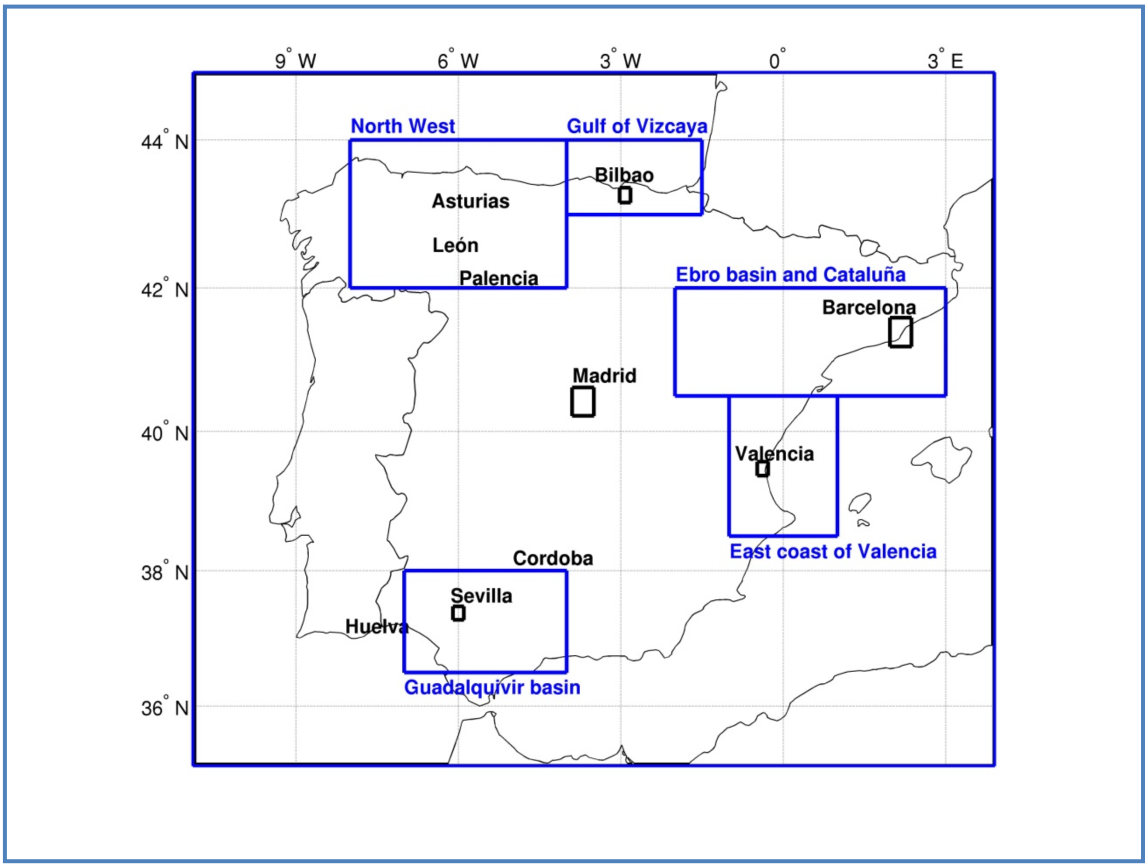

Figure S1. Map of the Iberian Peninsula including the location of the regions (Blue), the studied cities and the rest of places cited in the main text. Figure created by the authors using Matlab R213a

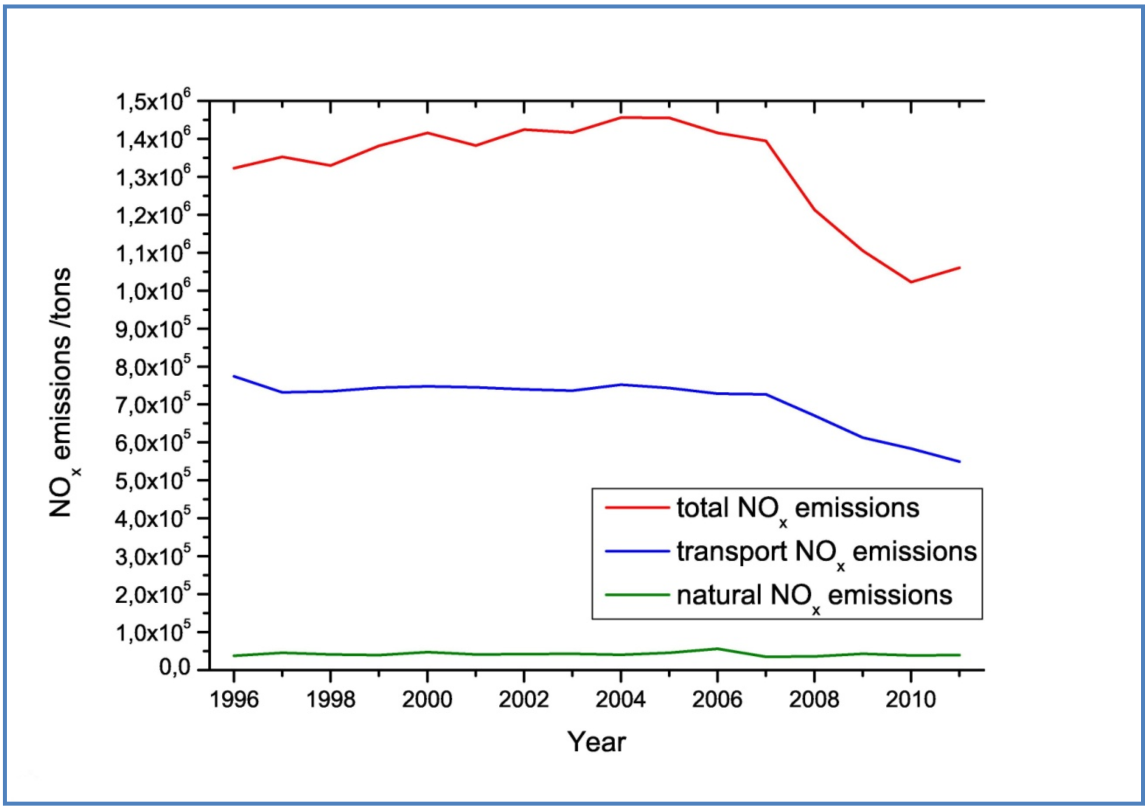

Figure S2. Spanish national emissions (MAGRAMA)[1] of NO_x.

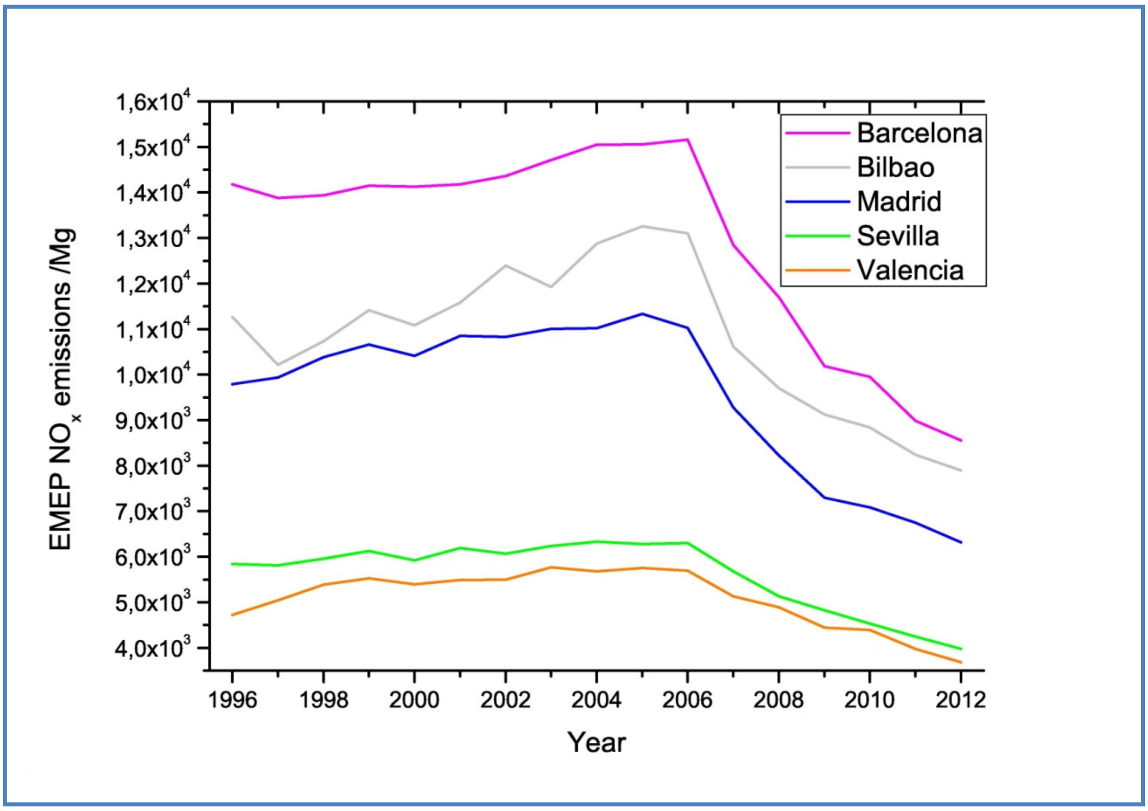

Figure S3. Emissions of NO_x as used in EMEP models for the five cities (CEIP)[2].

Geographic zone	Latitude	Longitude
**East coast of Valencia**	38.5 → 40.5	-1 → 1
**Ebro basin and Cataluna**	40.5 → 42	-2 → 3
**Madrid**	40.22 → 40.62	-3.9 → -3.5
**Sevilla**	37.28 → 37.48	-6.1 → -5.9
**Barcelona**	41.19 → 41.59	1.97 → 2.37
**Bilbao**	43.16 → 43.36	-3.02 → -2.82
**North-West**	42 → 44	-8 → -4
**Gulf of Vizcaya**	43 → 44	-4 → -1.5
**Gualdalquivir basin**	36.5 → 38	-7 → -4
**Valencia**	39.37 → 39.57	-0.48 → -0.28
**Spain**	36 → 44	-7 → 0
	42 → 44	-9.5 → -7
	39 → 43	0 → 3.5

Table S1. Coordinates of geographic zones included in table 1.

City	1996-2012		1996-2008		2008-2012	
	Slope / µg m^{-3} yr^{-1}	Error	Slope / µg m^{-3} yr^{-1}	Error	Slope / µg m^{-3} yr^{-1}	Error
Barcelona	-0.63*	0.13	-0.50	0.19	-0.89*	0.63
Bilbao	-1.09*	0.23	-1.44	0.31	-0.79*	0.47
Madrid	-1.41	0.21	-0.71	0.14	-4.31	0.94
Sevilla	-1.54**	0.18	-1.71**	0.33	-1.49	0.29
Valencia	-3.18***	0.23	-3.07***	0.42	-3.89	0.52
Average****	-1.48	0.13	-1.25	0.19	-2.89	0.39

Table S2. Slopes form the regression analysis for the different time periods.
*No data available for 2012.
**No data available for 1996.
***No data available for 1996 and 1997.
****Average of the five cities

Supplementary information references

1 MAGRAMA. Inventario Nacional de Emisiones de Contaminantes a la Atmósfera. Ministerio de Agricultura, Alimentación y Medio Ambiente del Gobierno de España (http://www.magrama.gob.es/es/calidad-y-evaluacion-ambiental/temas/sistema-espanol-de-inventario-sei-/). (2014, Date of access: 6/06/2014).

2 CEIP. Centre on Emission Inventories and Projections. (http://www.ceip.at/). (2014, Date of access: 8/07/2014).
